# Research on Companion-Based Forest Therapy and Its Physiological and Psychological Benefits to College Students

**DOI:** 10.3390/ijerph22071026

**Published:** 2025-06-27

**Authors:** Mei He, Yuan Hu, Xuan Dong, Jiarui Ma, Guangyu Wang

**Affiliations:** 1 Multidisciplinary Institute of Nature Therapy, Faculty of Forestry, University of British Columbia, 2424 Main Mall, Vancouver, BC V6T 1Z4, Canada; mei.he@ubc.ca (M.H.); irisdong0710@gmail.com (X.D.); jiaruima@student.ubc.ca (J.M.); 2 Jiangxi Academy of Forestry, 1629 Fenglin Road, Nanchang 330032, China

**Keywords:** emotional support, forest therapy, psychological resilience, social interaction, health management

## Abstract

With the growing pressures of modern society, physical and mental health issues have emerged as critical global concerns. Forest therapy (FT), a novel health management model that integrates natural environments with physical and mental healing, has gained increasing attention in recent years. However, mainstream FT approaches often overlook the psychological value of interpersonal interaction. Building upon traditional FT, this study proposes a new framework called companionship-based forest therapy (CBFT), which emphasizes the importance of emotional support within natural settings. CBFT is not intended as a replacement for conventional FT, but rather as an optimized approach that enhances its therapeutic effects by incorporating the element of companionship. This study aims to evaluate the physiological and psychological benefits of a novel intervention model—companion-based forest therapy (CBFT)—compared to conventional forest therapy models. Grounded in psychological theories and supported by empirical analysis, this study presents an applied framework of CBFT grounded in established psychological theories and validates its effectiveness through a comparative intervention involving 30 college students. Interpreted from the perspectives of ecological and humanistic psychology, the results indicate that CBFT significantly improves emotional regulation, reduces physiological stress responses, and enhances overall mental well-being. These findings highlight the value of social connection in FT practices and offer new directions for the development and application of forest therapy.

## 1. Introduction

Urbanization and high-paced lifestyles have increasingly contributed to mental health challenges, making emotional well-being a critical issue worldwide [1,2]. According to the World Health Organization (WHO), mental health encompasses not only the absence of illness but also emotional stability and adaptive functioning [3,4]. Persistent stressors such as long work hours, digital overload, and limited social interaction have been linked to rising rates of anxiety, depression, and cognitive fatigue. [5]. Additionally, aging populations are confronted with loneliness and diminished social engagement, which further aggravate health risks [6].

Despite technological advancements, many individuals remain in a persistent state of “sub-health”—a condition characterized by mild physiological and psychological imbalances. Symptoms such as poor sleep, low energy, and mood instability are common. As a result, conventional health solutions are proving insufficient, prompting the search for nature-based alternatives. Forest therapy (FT) has emerged as a promising intervention. Supported by growing empirical evidence, FT is known to reduce stress, enhance immune response, and improve emotional states [7,8]. Elements of forests, such as negative ions, fresh air, tranquil surroundings, and rich natural landscapes, effectively reduce physiological stress responses [9], enhance cognitive functions [10,11,12], and improve mental health, promoting overall recovery [13].

While FT highlights the restorative power of nature, it often overlooks the influence of social and emotional companionship on healing. Research indicates that human connection plays a vital role in managing stress and fostering resilience [14,15]. Consequently, relying solely on the healing properties of forest environments may not maximize their benefits, particularly for individuals dealing with psychological challenges and social isolation. This view aligns with that of Hartig et al. [16], who emphasized the synergistic relationship between natural settings and social context in promoting restorative experiences. Current FT research largely focuses on the experiences of individuals, prompting the proposal of the psychology-based “companionship-based forest therapy” (CBFT) model. This model integrates professional companions with the therapeutic effects of forest environments to enhance the physical and mental recovery of participants. CBFT emphasizes professional guidance and support, facilitating deeper interaction between individuals and natural environments to yield more profound psychological and physiological benefits.

Existing research on FT primarily explores its impacts on physical health, such as immunity and cardiovascular health. International studies have highlighted the positive effects of forest environments on stress reduction, immune enhancement, and emotional regulation [7,8]. While guided forest therapy has received attention, few studies isolate the structured role of a psychologically trained companion within brief FT sessions. Some scholars have proposed combining forest environments with psychological counseling and emotional companionship to create more effective wellness models, but this concept remains in its early stages [17]. Therefore, there is a significant research gap regarding the physiological and psychological benefits of CBFT.

To address this gap, this paper focuses on the effectiveness of CBFT, which is grounded in ecological and humanistic psychology. It investigates its impacts on physiological indicators (e.g., immunity, blood pressure, heart rate) and psychological health (e.g., emotional regulation, anxiety, depression), as well as its role in enhancing social support and well-being.

## 2. Core Concepts and Experimental Hypotheses

### 2.1. Conceptual Definitions

Companionship-based forest therapy (CBFT), as proposed in this study, extends the conventional FT model by incorporating structured social support into the therapeutic process. Rather than presenting CBFT as a separate category, it is conceptualized as a complementary enhancement to traditional FT. This approach underscores the role of human companionship—offered by trained guides or peers—in providing emotional encouragement, facilitating meaningful interaction, and strengthening participants’ sense of connection and security within nature. The model integrates Rogerian humanistic therapy principles (e.g., unconditional positive regard) and social buffering theory, asserting that companionship can significantly amplify FT’s psychological benefits.

CBFT is grounded in two complementary theoretical foundations: ecological psychology and humanistic psychology. The former emphasizes the restorative effects of nature on cognitive and emotional states, drawing from Kaplan’s Attention Restoration Theory and Ulrich’s Stress Recovery Theory. The latter introduces the importance of unconditional positive regard and empathetic interaction [18], which in CBFT is operationalized through structured companionship in nature. Together, these theories provide the basis for CBFT’s integration of environmental immersion and emotional support.

### 2.2. Experimental Hypotheses

Based on the aforementioned framework, this study formulates two primary hypotheses:

**Hypothesis 1:**  *Participants who receive CBFT will show greater improvements in physiological markers—specifically, heart rate variability (HRV)—and psychological indicators compared to those undergoing FT alone, without companionship. Participants receiving CBFT will demonstrate greater reductions in anxiety (STAI-S), perceived stress (PSS), and negative mood dimensions (depression, anger, fatigue) on the POMS, along with improved HRV (increased lnHF) and lower electrodermal activity (EDA), compared to NCFT.*

**Hypothesis 2:**  *Drawing on the principles of social support theory, CBFT is expected to elicit higher levels of subjective satisfaction and participant engagement than non-companionship-based forest therapy (NCFT).*

These hypotheses reflect the assumption that the combined effects of emotional support and environmental exposure enhance therapeutic outcomes to a greater extent than either element alone.

## 3. Materials and Methods

### 3.1. Research Design

To evaluate the effectiveness of companionship-based forest therapy (CBFT), a within-subject experimental design was implemented, involving a total of 30 undergraduate participants from the University of British Columbia (UBC). Despite the moderate sample size, this design aligns with precedents set by earlier forest therapy studies that produced reliable outcomes with similar participant numbers [13,19]. A statistical power analysis (α = 0.05, power > 0.8) confirmed the adequacy of the sample for detecting significant treatment effects. The primary goal was to compare the physiological and psychological outcomes between two types of interventions: CBFT (with companionship) and NCFT (standard FT without companionship). Each participant experienced both interventions in a randomized crossover format, with a minimum two-week washout period to mitigate carryover effects.

#### 3.1.1. Participants

Selection Criteria

Thirty students (15 males, 15 females; mean age = 21.97 ± 1.52 years) voluntarily enrolled in the study via campus-wide flyers and announcements (Table 1). No compensation was offered. Informed consent was obtained from all individuals, all participant data was anonymized using unique ID codes, stored on password-protected systems, and handled in compliance with UBC’s behavioral research ethics standards, and the study was approved by UBC’s Behavioral Research Ethics Board (Approval ID: H20-00621).

Eligibility criteria included:

- Absence of clinically diagnosed stress disorders or major depression;
- No history of substance abuse.

Exclusion criteria covered:

- Severe physical or mental health conditions;
- Pregnancy.

Each participant completed both types of FT interventions—CBFT and NCFT—with sessions conducted at least two weeks apart to prevent sequence bias.

#### 3.1.2. Intervention Procedures

(1) Experimental Group

The intervention involved a total of 30 participants (15 male, 15 female), who completed both CBFT and NCFT sessions in a crossover design. All sessions were scheduled by the research team between 10:00 a.m. and 12:00 p.m. on weekdays, under similar weather conditions. The CBFT group was guided by Mei He, a certified FT guide accredited by the Association of Nature and Forest Therapy Guides and a licensed psychological counselor certified by the Chinese Academy of Sciences. The participants engaged in a one-hour session of activities, including deep breathing exercises, forest walking, emotional sharing, and natural meditation (Table 2). The companion was the same licensed psychological counselor throughout, and followed a pre-scripted intervention protocol to ensure consistency in support and communication across all participants.

**Figure 1:**
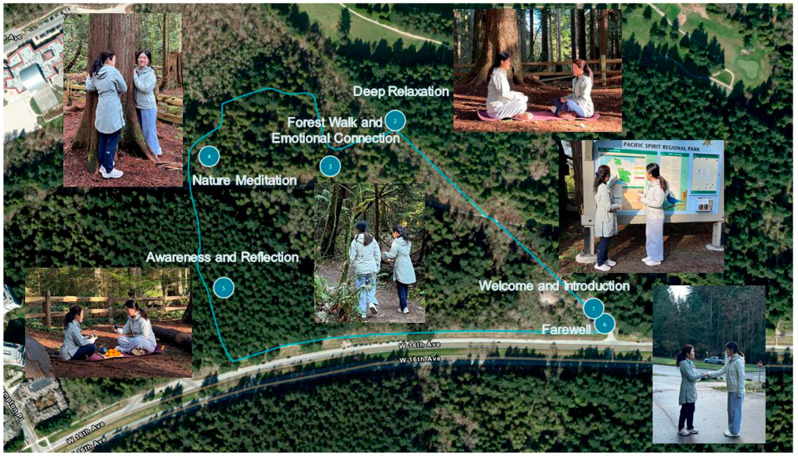
A depiction of the CBFT process.

The guide led participants along pre-planned routes and designated locations for therapeutic activities (Figure 1, Table 3), providing emotional support and guidance on relaxation techniques throughout the session.

(2) Control Group

The NCFT group involved participants conducting a one-hour FT session alone, while following the same route as that of the experimental group. The participants were free to choose activities such as walking, meditation, or sitting quietly, without any companionship or guidance provided during the session.

#### 3.1.3. Study Location

All interventions took place at Pacific Spirit Regional Park (PSRP), an urban forest reserve near UBC. The park offers over 85% forest coverage and a stable ecological environment, making it ideal for controlled FT research. Interventions were conducted during similar weather conditions (temperature: 18.5 ± 3.2 °C; humidity: 61.3 ± 7.8%), between 10:00 a.m. and 12:00 p.m. on clear days, to minimize external environmental variability.

**Figure 2:**
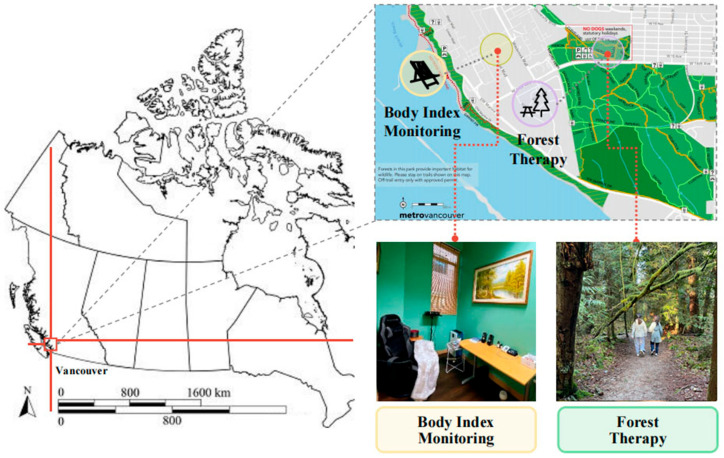
Location of the experiment.

Physiological and psychological data were collected and analyzed at the Forest Therapy Laboratory under the UBC Multidisciplinary Institute of Nature Therapy (Figure 2).

#### 3.1.4. Experimental Procedure

All participants received both intervention types in a crossover order. The randomized design helped balance any sequence effects, and the two-week interval minimized residual emotional or physiological influences. Each session was completed individually to reduce social influence from peers.

### 3.2. Data Collection and Measurement

To assess the impact of CBFT versus NCFT, both subjective psychometric tools and objective physiological indicators were employed. Data collection occurred before and after each intervention session, enabling within-subject comparison across conditions.

#### 3.2.1. Survey

Participants completed a structured questionnaire comprising three main sections:

Section 1**: Demographic Data**—basic information such as age, gender, and health status;

Section 2**: Stress Perception**—self-assessed ratings of daily stress and coping ability;

Section 3**: Interest in Forest Therapy Participation**—gauging willingness to engage in future FT activities.

Two validated psychological scales were utilized to capture the emotional and mental states of the participants:

- **State-Trait Anxiety Inventory (STAI-S)**: This instrument, developed by Spielberger et al. (1971) [20], measures state anxiety levels—transient emotional responses to stressors. The STAI-S was specifically chosen to assess anxiety fluctuations induced by the interventions [20].
- **Perceived Stress Scale (PSS)**: Created by Cohen et al. (1983) [21], the PSS evaluates the individual’s perception of stress in daily life and their perceived capacity to cope. The scale has been widely applied in evaluating the efficacy of stress reduction interventions and provides a comprehensive understanding of the participants’ general stress levels [21].

#### 3.2.2. Physiological Measurements

To capture the **physiological stress responses** associated with each therapy model, the following biometric indicators were collected:

- **Blood Pressure and Pulse Rate:**
  Systolic and diastolic blood pressure, along with heart rate, were measured using an upper-arm automatic monitor Model YE670A (Jiangsu Yuyue Medical Equipment Co., Ltd., Nanjing, China). Each participant underwent two consecutive readings after resting for 5 min in a seated position. If the systolic readings differed by more than 10 mmHg or diastolic readings by more than 6 mmHg, a third reading was taken. The mean of valid measurements was used for final analysis.
- **Heart Rate Variability (HRV):**
  HRV was evaluated using a portable electrocardiogram (ECG) device HeaLink-R211B (Healicom, Nanjing, China). R-R interval data were extracted and analyzed using the **Kubios HRV software (version 4.2.0, Kubios Oy, Kuopio, Finland)**. Two specific HRV metrics were examined:
  - *lnHF (log-transformed high-frequency power)*, reflecting parasympathetic nervous activity;
  - *lnLF/lnHF ratio*, indicating the balance between sympathetic and parasympathetic systems.

A rise in lnHF and a decrease in LF/HF were interpreted as signs of autonomic relaxation and reduced stress.

- **Electrodermal Activity (EDA):**
  EDA, which measures changes in skin conductance linked to sympathetic nervous activity, was recorded using a Biopac MP150 system (Goleta, CA, USA) with EDA100C and ECG100C modules. Electrodes were attached to participants’ index and middle fingers. EDA values were averaged over 2-min intervals, and lower values were taken as indicators of decreased physiological arousal and stress [1].

#### 3.2.3. Psychological Measurements

The Profile of Mood States (POMS) is a reliable and valid scale for measuring psychological distress [22]. It is a standard tool for evaluating the effectiveness of psychotherapy. Similar to the STAI scale, if emotional companionship can promote emotional regulation, the CBFT group should have lower scores on the POMS negative emotion subscale and higher scores on vitality. The POMS developed by [23] and revised by [24] was adopted in this study. The scale provides a comprehensive reflection of the emotional status of participants and has high reliability. The simplified POMS consists of 40 items, including seven emotional state indicators: tension–anxiety (T), depression–dejection (D), anger–hostility (A), fatigue–inertia (F), confusion–bewilderment (C), vigor (V), and self-esteem (S). The first five items represent negative emotions (negative scale, with higher scores indicating worse emotions), while the last two items represent positive emotions (positive scale, with higher scores indicating more positive emotions). Each indicator reflects the level of a specific emotion reported by the participant. The Total Mood Disturbance (TMD) score is calculated by subtracting the sum of the two positive emotional scores from the sum of the five negative emotional scores, and then adding a constant of 100. A higher TMD score indicates a more negative emotional state.

### 3.3. Data Analysis Methods

To evaluate the impact of the two forest therapy models—CBFT and NCFT—on participants’ physiological and psychological states, a combination of **descriptive and inferential statistical methods** was applied.

#### 3.3.1. Descriptive Statistics

Initial data exploration involved calculating basic descriptive statistics. Categorical variables were summarized using frequencies and proportions, while continuous variables were expressed as **means ± standard deviations** (SD). These summaries provided a foundational understanding of participant characteristics and baseline measurements.

#### 3.3.2. Inferential Analysis

To compare pre- and post-intervention outcomes within and between groups, the following statistical tests were employed:

- **Paired Sample *t*-tests**: Applied to examine within-subject differences before and after the interventions in physiological and psychological metrics (e.g., blood pressure, heart rate, HRV, STAI-S, PSS, and POMS scores).
- **Wilcoxon Signed-Rank Test**: For variables that did not meet normality assumptions, this non-parametric alternative to the paired *t*-test was utilized, particularly in analyzing changes in POMS dimensions.

All data analyses were conducted using **SPSS Version 20.0 (IBM Corp., Armonk, NY, USA)**. A ***p*-value less than 0.05** was considered statistically significant. For multiple comparisons, **Holm’s correction** was applied to adjust for family-wise error rates and reduce the likelihood of Type I errors.

The outcome variables of interest included:

- **Physiological indicators**: SBP, DBP, heart rate, HRV (lnHF, lnLF/lnHF), and EDA;
- **Psychological measures**: POMS (subscales and TMD), STAI-S, and PSS.

These analytical procedures enabled a rigorous evaluation of CBFT’s effectiveness compared to conventional forest therapy.

## 4. Research Results

### 4.1. Theoretical Analysis Results

The theoretical underpinnings of CBFT rest on two complementary psychological frameworks: **Ecopsychology** and **Humanistic Psychology**. The former posits that human well-being is intricately linked to environmental exposure—especially interactions with natural ecosystems. Forest environments offer multisensory stimuli that aid emotional regulation and cognitive recovery. CBFT enhances these effects by incorporating structured companionship, where the presence of a guide helps participants engage more deeply and derive psychological comfort.

Meanwhile, humanistic theory emphasizes the importance of unconditional positive regard, emotional acceptance, and the pursuit of self-actualization. CBFT operationalizes these principles by offering empathic support within the therapeutic setting. Companions not only facilitate emotional expression but also foster feelings of acceptance and psychological safety, which contribute to mental healing and personal growth. The combined impact of nature and emotional support forms a multidimensional recovery mechanism.

### 4.2. Survey Results

Table 4 summarizes the participants’ stress self-assessment results, including percentage distribution and average scores. The pre-experiment survey data indicate that the participants were indeed experiencing stress, with 84% of respondents reporting that they felt stressed in their daily life and studies. Only a small portion of the respondents (14%) disagreed or strongly disagreed with this assessment. It is noteworthy that, under stressful conditions, only 16% of the respondents reported being able to self-regulate, while 77% stated that they could not manage stress well. Furthermore, the majority of the respondents (86%) expressed a desire for external help when facing stress. Additionally, the post-experiment survey results showed that a considerable portion of the participants (80%) expressed a willingness to participate in CBFT again, while only 56% were willing to engage in NCFT again.

CBFT provides high-quality emotional companionship through emotional support in social support theory and interaction with nature. The companion, in the activity, is not only a guide but also an emotional supporter. They help participants relax, relieve stress, and enhance emotional stability and a sense of belonging through positive interactions and care, thereby increasing their satisfaction and engagement in the activity. Social support theory suggests that, when participants feel supported and understood, their emotional regulation ability and engagement in the activity are significantly enhanced. Therefore, participants in the CBFT group experience higher satisfaction and stronger involvement, showing more emotional openness and engagement in the activity.

### 4.3. Changes in Physiological Indicators

The overall results are summarized in Table 5. For the CBFT group, the average SBP after the accompanied FT program (106.37 ± 14.16 mm Hg) was significantly lower (*p* < 0.001) than the baseline SBP (119.40 ± 9.70 mm Hg). Similarly, the average DBP after the accompanied FT program (72.63 ± 12.31 mm Hg) was also significantly lower (*p* = 0.006) than the baseline DBP (80.43 ± 10.60 mm Hg). Moreover, the average pulse rate measured after the accompanied FT program (71.70 ± 10.14 bpm) showed a significant decrease (*p* = 0.016) as compared to the baseline (77.83 ± 11.04 bpm).

For the NCFT group, the average SBP after the unaccompanied FT program (110.50 ± 9.63 mm Hg) was also significantly lower (*p* = 0.00058) than the baseline SBP (119.23 ± 8.73 mm Hg), but the decrease was smaller than that for the CBFT group (106.37 ± 14.16 mm Hg). Compared to that of the CBFT group, the decrease in DBP for the NCFT group was less significant (*p* = 0.022), with the baseline DBP (80.33 ± 10.63 mm Hg) dropping to 74.63 ± 10.44 mm Hg after the unaccompanied FT program. Furthermore, when comparing the pulse rates between the two groups, the NCFT group exhibited only a slight decrease in pulse rate (73.70 ± 10.23 bpm) as compared to the baseline (76.53 ± 10.77 bpm), and this difference was not statistically significant (*p* = 0.15).

The participants in both the CBFT group (*p* < 0.001) and the NCFT group (*p* < 0.001) showed a significant increase in lnHF after the FT. However, the increase for the CBFT group (6.99 ± 1.07 ln (ms^2^)) over the baseline (5.23 ± 0.82 ln (ms^2^)) was larger than that for the NCFT group (6.76 ± 0.75 ln (ms^2^)) over the baseline (5.51 ± 0.72 ln (ms^2^)). Both groups also showed a significant decrease in lnLF/lnHF (*p* < 0.0001 for the CBFT group, *p* = 0.007 for the NCFT group), with a more pronounced decrease in the CBFT group.

Both groups exhibited a decrease in EDA, but the participants in the CBFT group (*p* = 0.0004) experienced a greater reduction from the baseline than those in the NCFT group (*p* = 0.03). The participants in the CBFT group decreased from a baseline of 1.48 ± 1.01 μS to 0.65 ± 0.74 μS, while those in the NCFT group decreased from a baseline of 1.49 ± 0.96 μS to 1.04 ± 0.82 μS.

Restorative environment theory suggests that natural environments have restorative functions, helping individuals recover psychological and physiological health, reduce stress, and promote physical and mental relaxation. According to this theory, exposure to natural environments (such as nature walks and meditation in forest therapy) helps enhance parasympathetic nervous system activity, reduces overactivation of the sympathetic nervous system, and thus promotes emotional regulation and physiological recovery. When individuals enter a natural environment, the richness of sensory experiences and restorative elements help reduce anxiety and tension, leading to improved heart rate variability. CBFT, through interaction with the natural environment, guides participants into a relaxed state, reducing physiological stress responses, as reflected in lower EDA. Activities such as meditation and forest walks in CBFT help participants enter a state of relaxation, which aids in regulating blood pressure and relieving cardiovascular stress. By guiding participants to relax their bodies and focus their attention on the natural environment, CBFT gradually alleviates external pressures and internal tension. This relaxed state reduces sympathetic nervous system activation, slows the heart rate, resulting in a lower heart rate. Restorative environment theory points out that the richness and restorative characteristics of the environment promote relaxation, leading to a decrease in heart rate. These effects of restorative environments help reduce physiological stress responses, bringing heart rates back to normal and stable levels [25].

Restorative elements in natural environments (such as compatibility, sensory richness, attentiveness, and distance from everyday stress) can effectively reduce overactivation of the sympathetic nervous system, promote parasympathetic nervous system activity, and thereby facilitate emotional and physiological recovery. These changes in physiological indicators suggest that CBFT, through deep interaction with the natural environment, helps individuals restore psychological and physiological balance, alleviate stress, and enhance overall health.

### 4.4. Changes in Psychological Condition

Both CBFT and NCFT interventions led to significant improvements in participants’ psychological conditions, specifically in reducing self-reported anxiety and perceived stress levels. Statistical analysis revealed highly significant decreases in anxiety and stress scores for both groups (*p* < 0.001). Notably, the CBFT group demonstrated greater reductions in these measures compared to the NCFT group, indicating a stronger effect of companionship-based forest therapy on psychological well-being. Detailed pre- and post-intervention data are provided in Table 6.

**Figure 3:**
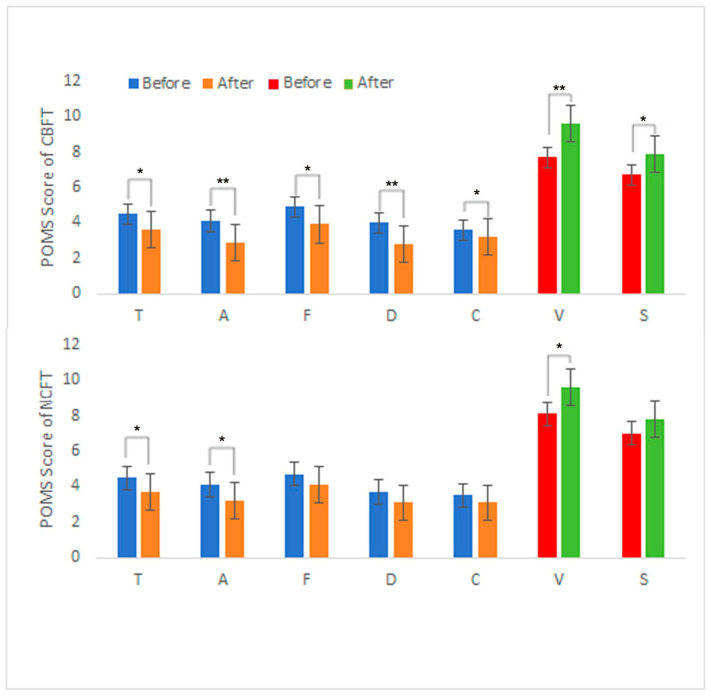
POMS results of the participants before and after the FT programs. Note: *T*: tension–anxiety; *D*: depression–dejection; *A*: anger–hostility; *F*: fatigue–inertia; *C*: confusion–bewilderment; *V*: vigor; *S*: self-esteem. Significant differences are marked with * *p* < 0.05 and ** *p* < 0.01.

**Figure 4:**
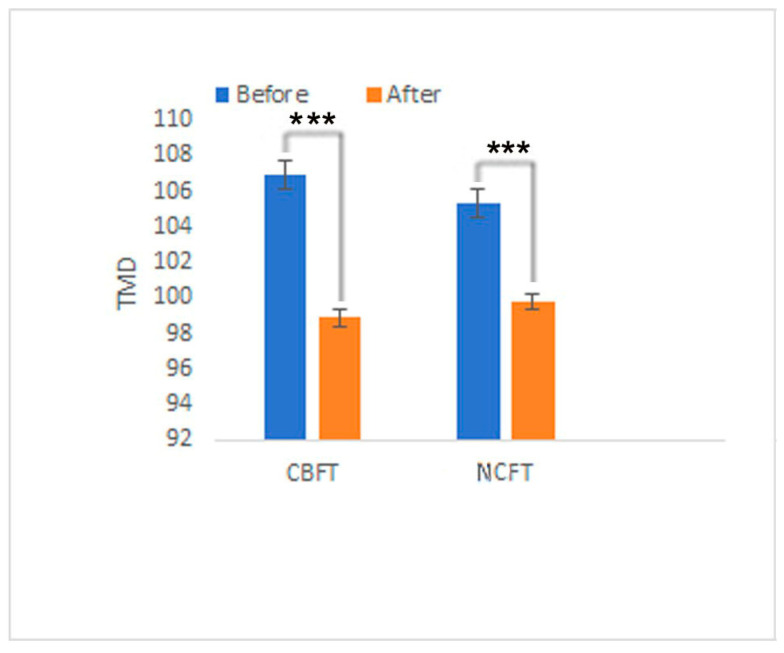
The TMD scores of the participants before and after the FT programs. Note: Significant differences are marked with *** *p* < 0.005.

The participants in both the CBFT and NCFT groups exhibited a positive emotional shift, as determined by the POMS scale assessment (Figure 3): positive emotion scores significantly increased, while negative emotion scores significantly decreased. For all participants, after each FT program, the average POMS scores for negative mood states (T, A, F, D, C) all decreased, while those for positive mood states (V, S) all increased. However, in the CBFT group, the decreases in T, F, and C were significant (*p* < 0.05), and the decreases in A and D were highly significant (*p* < 0.01). Regarding positive emotions, S showed a highly significant increase (*p* < 0.01), and V showed a significant increase (*p* < 0.05). In contrast, in the NCFT group, only the negative emotions T and A showed significant decreases (*p* < 0.05), V showed a significant increase, and there were no significant changes in the other emotions. Regarding the TMD scores (Figure 4), the CBFT group exhibited a great decrease, with scores decreasing from 106.9 to 98.9, while the scores of the NCFT group only decreased from 105.3 to 99.8.

In CBFT, the companion, through unconditional positive regard from humanistic psychology, creates an environment free from judgment and pressure, allowing participants to freely experience and express themselves. This process helps participants release negative emotions such as anxiety and depression, significantly improving their emotional state. The significant decrease in scores for negative emotions such as “anxiety” and “depression” on the POMS and STAI scales reflects how participants gradually alleviate these negative emotions through unconditional acceptance and understanding in CBFT. Humanistic psychology asserts that in such an accepting environment, individuals can effectively cope with and manage emotions, reducing levels of anxiety and depression. Self-actualization, one of the core goals of humanistic psychology, is reflected in the reduction of stress levels on the PSS scale, indicating that participants enhanced their self-regulation ability through CBFT and are better equipped to cope with daily life stress. The decrease in TMD (Total Mood Disturbance) reflects the overall improvement in participants’ emotional states. The reduction in anxiety scores on the STAI scale and the lowered perceived stress levels on the PSS scale indicate that participants achieved significant progress in the multidimensional process of self-regulation.

CBFT, through unconditional positive regard, self-actualization, positive emotional regulation, and holistic healing, significantly improves participants’ emotions and stress perception, promoting self-recovery in the natural environment. Through this process, participants enhance self-acceptance, self-identity, and emotional regulation skills, leading to reductions in negative emotions such as anxiety and depression on the POMS, STAI, and PSS scales, as well as alleviation of stress perception and emotional distress.

## 5. Discussion

### 5.1. Psychological Mechanisms of CBFT

CBFT is a health promotion approach that positively impacts individuals’ mental and physical health through interaction with nature and emotional support. The psychological mechanisms of CBFT include emotional regulation, stress management, social support, and self-growth. The core mechanism is the restorative effect of the natural environment provided by ecological psychology, combined with emotional support and unconditional positive regard from humanistic psychology, offering individuals a comprehensive space for psychological recovery.

Natural environments, especially forests, trigger relaxation responses through sensory stimuli (visual and auditory), significantly reducing psychological stress and enhancing psychological resilience [16,26]. As Hartig and colleagues noted, restorative outcomes in nature are closely tied to the presence of supportive social interactions, which further enhance psychological benefits. According to attention restoration theory and stress reduction theory, natural environments can improve mental health by reducing cognitive load and negative emotions, thus fostering a harmonious relationship with nature. This effect is particularly significant in CBFT, as the presence of a companion provides additional feelings of safety and belonging, which enhance comfort and help the body quickly enter a relaxed state [25]. The role of companionship serves as an emotional buffering mechanism in psychology [18]. Companions offer emotional care to help participants express emotions and relieve stress, thus forming emotional bonds in the natural environment, reducing the negative effects of loneliness and social isolation, and strengthening individuals’ sense of identity and social belonging. Emotional resonance also influences individuals by enhancing their sense of happiness [27]. Psychology emphasizes that the perception of meaning is an important component of health. In CBFT, individuals change their perception of stressful events or life meaning through interaction with nature and communication with companions. By experiencing positive emotions and life meaning, individuals can improve their mental state and enhance their psychological resilience. CBFT improves emotional and behavioral patterns by combining the natural environment and social support to significantly reduce anxiety and depression levels, ultimately promoting psychological healing. Participants reported greater awareness of stress triggers and emotional responses, although structured behavioral change was not an explicit focus of this study.

CBFT focuses on connection with nature, support from companionship, cognitive restructuring, and emotional regulation. By integrating sensory healing, emotional support, and cognitive guidance, CBFT comprehensively promotes participants’ mental health and well-being. Rather than proposing a novel psychological theory, CBFT adapts existing frameworks—particularly the social buffering hypothesis and attention restoration theory—to construct a dual pathway that integrates structured social support within a forest therapy context. This mechanism is applicable in various contexts, including stress management, psychological rehabilitation, and individual growth.

### 5.2. Comparison of Physiological Benefits

Natural elements in the forest environment, such as greenery, trees, fresh air, and birdsong, are considered to have a positive impact on individuals’ physiological healt [28,29,30]. The effects of forest environments on the cardiovascular system have also been widely studied. FT helps individuals reduce stress by lowering heart rate and blood pressure, thereby improving cardiovascular health. Research has shown that exposure to natural environments, especially forests, green spaces, and other natural landscapes, can significantly reduce sympathetic nervous activity and blood pressure while activating the parasympathetic nervous system, leading to a decrease in heart rate. Physiologists believe that this change is primarily related to the psychological relaxation effect brought by the natural environment, which reduces psychological stress responses and further influences autonomic nervous system activity [19,31,32].

Blood pressure is jointly regulated by the sympathetic and parasympathetic nervous systems. Sympathetic nervous activity increases blood pressure, while parasympathetic nervous activity decreases it [33]. The FT program administered in this study was found to lower systolic and diastolic blood pressure by reducing sympathetic nervous activity, which is consistent with previous studies measuring stress responses and autonomic nervous activity in healthy young individuals using various methods [34]. Most past studies have shown that participants’ pulse rates significantly decreased after entering a forest, indicating that forests have restorative effects on health [35]. In the present study, after the accompanied FT program, significant increases in HF values and decreases in LF/HF values were recorded, indicating that the forest environment can induce autonomic nervous relaxation and improve cardiovascular health [22]. The forest environment promotes parasympathetic nervous activity, suppresses sympathetic nervous activity, and leads to a decrease in EDA [1].

The physiological results also indicate that both FT programs administered in this study had positive effects on the participants’ autonomic nervous system activity, lowering their blood pressure and heart rate. An analysis of HRV and EDA data also showed that, after FT, the parasympathetic nervous system was activated and the sympathetic nervous system was inhibited, indicating that FT indeed promoted physiological relaxation in the participants.

Although the participants’ blood pressure and heart rate significantly decreased, their HF increased, their LF/HF decreased, and their EDA decreased after both FT programs, the positive changes in SBP, DBP, heart rate, LF/HF, and EDA were more pronounced in the CBFT group. This indicates that the effects of CBFT were great, resulting in lower stress levels and better cardiovascular health. Additionally, the participants in the CBFT group exhibited stronger parasympathetic nervous activity than those in the NCFT group.

CBFT provides great physiological stress relief, as the presence of a companion during the therapy offers emotional support. The encouragement, guidance, and social interaction from the companion help enhance participants’ psychological sense of security and belonging, making it easier for them to enter a relaxed state and promoting physiological relaxation. In contrast, while the natural environment itself has a positive effect on physiological relaxation in NCFT, the lack of external support and interaction may prevent participants from fully relaxing during the activity.

### 5.3. Comparison of Psychological Benefits

From a psychological perspective, all participants showed significant improvement on self-assessment scales for anxiety and stress, with companionship playing a key role in alleviating anxiety and depressive symptoms. However, the CBFT group exhibited a more pronounced decrease in the SA scale and PSS scores than the NCFT group.

Additionally, after both FT programs, the average scores for negative emotional states on the POMS (T, A, F, D, C) decreased, while the average scores for positive emotional states (V, S) increased. Regarding the participants in the CBFT group, the scores for the emotional state indicators T, A, F, D, and C, as well as the TMD score, significantly decreased, with A and D showing a very significant decrease, while the scores for V and S increased, with V showing a very significant increase. However, for the participants in the NCFT group, only the scores for the state indicators T and A, as well as the TMD score, showed significant decreases, and only the score for V showed a significant increase.

In the CBFT group, the presence of a companion played an important role in alleviating anxiety and depressive symptoms. In the natural environment, social support and emotional interaction help participants feel psychological safety and a sense of belonging, thus enhancing their self-awareness and reducing anxiety and depression levels. Through interaction with others, participants not only relax physically and mentally, but also receive emotional guidance and cognitive feedback. This process helps participants develop a more positive self-evaluation and healthy emotional regulation strategies. Studies have also shown that sharing the tranquility and beauty of nature with others can induce stronger feelings of happiness and satisfaction, effectively relieving negative emotions.

Although NCFT was found to have some effect on emotional regulation due to the natural environment itself, the lack of interaction with a companion and the absence of guidance may cause participants to be more likely to fall into negative emotional rumination, especially when alone. Symptoms of depression and anxiety may not be effectively alleviated, and the lack of support makes it difficult for participants to identify and process their emotions. Feelings of loneliness and a lack of support may slow down the emotional recovery process, and some participants may feel isolated and helpless.

### 5.4. Integrated Application of Dual Mechanisms in CBFT’s Psychological and Physiological Effects

This section interprets CBFT’s effectiveness through the lens of two well-established psychological frameworks, without proposing a new theoretical model. Rather, it explores how the interaction between psychological and physiological mechanisms is operationalized in the CBFT setting.

#### 5.4.1. Psychological-Physiological Interaction Effect

Both psychological and physiological research have shown that there is a close interaction effect between a person’s psychological state and physiological responses. Psychological stress and negative emotions influence physiological processes through the neuroendocrine system, and, conversely, improvements in physiological states can also have a positive impact on mental health. During CBFT, the natural environment helps individuals reduce their psychological burden through its restorative effects, while the emotional support and companionship provided by professionals enhance psychological safety and reduce feelings of stress. This psychological relief, via the regulation of the autonomic nervous system, improves cardiovascular health, lowers stress hormones, and ultimately promotes the recovery of physical health.

#### 5.4.2. Psychological–Physiological Positive Feedback Mechanism

From a physiological perspective, the physiological effects of the forest environment, such as lowering blood pressure, reducing heart rate, and regulating immune functions, in turn promote mental health. Lowered stress hormone levels not only alleviate anxiety but also enhance an individual’s ability to regulate their emotions. When physiological stress responses are effectively alleviated, an individual’s emotions become more stable, psychological stress is released, and a positive cycle is established.

An individual’s emotional and psychological states are regulated through the support of companions and the healing effects of the natural environment, which then affect their physical health. Positive emotions and good psychological states can regulate the neuroendocrine system, reduce stress responses, and promote the body’s self-repair. Psychological relaxation and a sense of well-being can lower blood pressure, slow the heart rate, and improve sleep quality, thereby further enhancing overall physical health.

### 5.5. Limitations and Future Research Directions

Several limitations of this study should be acknowledged. First, a total of 30 participants from a single university were enrolled, which limits generalizability. Gender interaction effects were not separately analyzed due to sample size. Second, the intervention period was relatively short, which might not fully capture the sustained physiological and psychological benefits of the therapy. Third, the study lacked long-term follow-up data, making it difficult to assess the persistence of the observed effects over time. Lastly, while the study focused on companion-based forest therapy, it did not explore the potential differential effects brought about by different types of “companions”. In addition, the two-week interval helped reduce the interference of intervention order effects; however, changes in the external environment may still have influenced the results. Future research should aim to address these limitations by including larger and more diverse samples, extending the intervention duration, incorporating long-term follow-ups, and comparing the effects of various companion types. Future research should also draw on methodologies developed by researchers at SLU (Sweden) and the University of Copenhagen, where innovative nature-based interventions have been applied to urban populations.

## 6. Conclusions

As an integrated health management model that combines the natural environment with professional psychological companionship, CBFT draws on the theoretical frameworks of ecological psychology and humanistic psychology. Based on the natural forest environment, CBFT provides participants with deep psychological and emotional support, significantly promoting physical and mental well-being. By comparing the effects of accompanied FT with unaccompanied FT, the following conclusions can be drawn: (1) CBFT has great effects on relieving stress physiologically, reducing heart rate and blood pressure, maintaining cardiovascular health, enhancing parasympathetic nervous activity, and improving autonomic nervous balance. While NCFT also has some physiological relaxation effects, its impact is limited due to the lack of support from companionship. (2) CBFT is more effective in alleviating participants’ anxiety, depression, and other negative emotions, and it promotes emotional regulation and self-awareness. NCFT has certain limitations in emotional regulation and may not fully achieve the expected psychological adjustment effects. (3) The CBFT model enhances participants’ sense of belonging and willingness to participate by providing social support and interaction, while NCFT lacks such social support, leading to a significantly reduced desire for participants to engage again.

In summary, compared to NCFT, CBFT not only promotes physical recovery through the positive impact of the natural environment on physical health, but also helps individuals regulate emotions, alleviate stress, and achieve personal growth through psychological mechanisms. It provides a more comprehensive and effective health-promoting effect on both the physiological and psychological levels. Due to the combination of these factors, CBFT is an effective method for physical and mental treatment, promoting overall health at multiple levels. Therefore, future health and wellness activity designs should more strongly consider the element of companionship, in which the CBFT model demonstrates greater potential and advantages. CBFT was associated with greater improvements in physiological and psychological outcomes compared to NCFT, although findings should be interpreted cautiously due to sample size and limited population diversity.

## Figures and Tables

**Table 1:** Demographic information on the participants.

Parameter	Mean ± Standard Deviation		
	All Participants	Male	Female
Number of samples	30	15	15
Age (years)	21.97 ± 1.52	22.20 ± 1.47	21.73 ± 1.53
Height (cm)	163.44 ± 10.24	169.43 ± 7.71	157.45 ± 8.87
Weight (kg)	61.48 ± 7.67	65.47 ± 3.24	57.49 ± 8.67
BMI (kg/m^2^)	22.99 ± 1.890	22.93 ± 2.12	23.06 ± 1.64

BMI: Body mass index.

**Table 2:** Structure and objectives of the FT intervention session.

Activity Segment	Objective	Duration (min.)
1: Welcome and Introduction	Build initial rapport, understand participants’ emotional state, and reduce anxiety through verbal interaction.	5
2: Deep Relaxation	Use breathing and sensory grounding to reduce physiological arousal.	10
3: Forest Walk and Emotional Connection	Encourage emotional connection with nature and the companion, enhancing social support.	20
4: Nature Meditation	Promote mindfulness and inner calm through guided nature-based meditation.	10
5: Awareness and Reflection	Facilitate emotional expression and self-awareness through group sharing.	10
6: Farewell	Help participants return smoothly to daily life with body stretching and closure.	5

**Table 3:** Locations and elevations of the FT sessions.

Stop	Activity	Location	Elevation (ASL)
1(6)	Welcome and Introduction (Farewell)	49°25′88.3″ N 123°22′20.7″ W	99 m
2	Deep Relaxation	49°26′10.7″ N 123°22′56.7″ W	106 m
3	Forest Walk and Emotional Connection	49°26′06.6″ N 123°22′71.9″ W	125 m
4	Nature Meditation	49°26′06.7″ N 123°22′94.5″ W	136 m
5	Awareness and Reflection	49°25′93.7″ N 123°22′90.2″ W	115 m

Notes: Stop refers to specific GPS-logged rest points used during therapy.

**Table 4:** Summary of participants’ stress self-assessment responses (*n* = 30).

Assessment Item	Agree (%)	Neutral (%)	Disagree (%)	Mean Score (1–5)
“I often feel stressed in daily life and study.”	84%	2%	14%	4.3 ± 0.7
“I can manage and regulate stress well.”	16%	7%	77%	2.1 ± 0.9
“I need external support when I’m under stress.”	86%	7%	7%	4.4 ± 0.6
“I am willing to participate in CBFT again.”	80%	10%	10%	4.2 ± 0.5
“I am willing to participate in NCFT again.”	56%	17%	27%	3.5 ± 0.8

**Table 5:** Physiological changes of the participants before and after the FT programs.

Measurement Time	Group	SBP (mmHg)		DBP (mmHg)		Pulse Rate (bpm)		lnHF [ln (ms^2^)]		lnLF/lnHF		EDA (μS)	
		Mean ± SE	*p*-Value	Mean ± SE	*p*-Value	Mean ± SE	*p*-Value	Mean ± SE	*p*-Value	Mean ± SE	*p*-Value	Mean ± SE	*p*-Value
Before	CBFT	119.40 ± 9.70	-	80.43 ± 10.60	-	77.83 ± 11.04	-	5.23 ± 0.82	-	1.19 ± 0.21	-	1.48 ± 1.01	
	NCFT	119.23 ± 9.83	-	80.33 ± 10.63	-	76.53 ± 10.77	-	5.51 ± 0.72	-	1.13 ± 0.07	-	1.49 ± 0.96	
After	CBFT	106.37 ± 14.16	<0.001 ***	72.63 ± 12.31	0.006 **	71.70 ± 10.14	0.016 *	6.99 ± 1.07	<0.001 ***	0.98 ± 0.19	<0.0001 ***	0.65 ± 0.74	0.0004 ***
	NCFT	110.50 ± 9.63	0.00058 ***	74.63 ± 10.44	0.022 *	73.70 ± 10.23	0.15	6.76 ± 0.75	<0.001 ***	1.02 ± 0.15	0.007 **	1.04 ± 0.82	0.03 *

Notes: *n* = 30; SBP: systolic blood pressure, DBP: diastolic blood pressure, SE: standard error. Ln: natural logarithm (the spectral power data were log-transformed), LF: power in the low-frequency range, HF: power in the high-frequency range, EDA: electrodermal activity; *, **, and *** respectively represent *p* < 0.05, 0.01, and 0.001 as determined by a paired *t*-test (one-sided) with Holm correction.

**Table 6:** Participants’ psychological changes before and after the FT programs.

Measurement Time	Group	SA		PS	
		Mean ± SE	*p*-Value	Mean ± SE	*p*-Value
Before	CBFT	39.97 ± 3.75	-	23.77 ± 4.76	-
	NCFT	38.67 ± 3.36	-	23.53 ± 4.64	-
After	CBFT	29.33 ± 3.33	<0.001 ***	15.10 ± 2.40	<0.001 ***
	NCFT	31.23 ± 4.00	<0.001 ***	18.53 ± 3.81	<0.001 ***

Notes: *n* = 30; PS: perceived stress, SA: state anxiety, SE: standard error; *** represents *p* < 0.001 as determined by a paired *t*-test (one-sided) with Holm correction.

## Data Availability

The original contributions presented in this study are included in the article. Further inquiries can be directed to the corresponding author(s).
